# Sex dimorphism in isoproterenol-induced cardiac damage associated neuroinflammation and behavior in old rats

**DOI:** 10.3389/fnagi.2022.854811

**Published:** 2022-07-22

**Authors:** Kata Tóth, Tamás Oroszi, Eddy A. van der Zee, Csaba Nyakas, Regien G. Schoemaker

**Affiliations:** ^1^Department of Neurobiology, Faculty of Science and Engineering, GELIFES, University of Groningen, Groningen, Netherlands; ^2^Research Center for Molecular Exercise Science, University of Physical Education, Budapest, Hungary; ^3^Behavioral Physiology Research Laboratory, Health Science Faculty, Semmelweis University, Budapest, Hungary; ^4^Department of Mobility, University Medical Center Groningen, Groningen, Netherlands

**Keywords:** advanced age, sex dimorphism, isoproterenol, cardiac damage, short-term memory, open field exploration, neuroinflammation, brain derived neurotrophic factor

## Abstract

Acute cardiac damage can be induced by isoproterenol injections in animals. The associated inflammatory response could be reflected in the brain as neuroinflammation, with potential consequences for brain function and behavior. Although cardiac responses are reported age and sex-related, for neuroinflammation and brain function this is virtually unknown. Therefore, cardiac damage and its consequences for neuroinflammation, brain function and behavior were compared in aged male and female rats. Wistar rats of 24 months of age were treated with isoproterenol (ISO, twice s.c.) or saline. Four weeks after injections, exploratory behavior and short-term memory were tested. Then, rats were sacrificed. Hearts were collected to measure cardiac damage. Brain tissue was collected to obtain measures of neuroinflammation and brain function. In male-, but not in female rats, ISO induced significant cardiac damage. Accordingly, mortality was higher in males than in females. Baseline hippocampal microglia activity was lower in females, while ISO induced neuroinflammation in both sexes, Hippocampal brain-derived neurotrophic factor expression appeared lower in females, without effects of ISO. In the open field test, ISO-treated males, but not females, displayed anxiety-like behavior. No effects of ISO were observed on short-term memory in either sex. In conclusion, sex dimorphism in effects of ISO was observed for cardiac damage and open field behavior. However, these effects could not be related to differences in hippocampal neuroinflammation or neuronal function.

## Introduction

Acute sympathetic stress is associated with overactivation of the sympathetic nervous system, evoking inflammation and increased cytokine production in the heart, resulting in cardiac damage and consequently cardiac dysfunction (Alemasi et al., 2019). Although these cardiac effects are extensively investigated, effects on other organs, including the brain, are far less known. However, the peripheral inflammatory response could well be reflected in the brain as neuroinflammation (Quan, 2014; Gouweleeuw et al., 2017), and depending on the affected brain area, may result in behavioral changes associated with mood and cognition. Moreover, sex-differences were observed for depression as well as cognitive decline (Eastwood et al., 2012; Deckers et al., 2017). Finally, mental problems in cardiovascular disease are not harmless, as they are associated with increased morbidity and mortality (Meijer et al., 2013).

Acute administration of high dose of beta-receptor agonist isoproterenol (ISO) was used to mimic the clinical condition of acute sympathetic stress (Beznak and Hacker, 1964; Nichtova et al., 2012; Adamcova et al., 2019). ISO, administered twice with 24h in between, evoked inflammation and increased cytokine production resulting in cardiac fibrosis (Adamcova et al., 2019; Alemasi et al., 2019), that over a period of weeks developed into left ventricular hypertrophy and dilatation, and ultimately heart failure (Nichtova et al., 2012). In young male rats, this process was associated with reduced exploratory behavior (Tkachenko et al., 2018), depressive-like behavior (Hu et al., 2020) and cognitive decline (Ravindran et al., 2020).

Cardiac effects of ISO were shown dependent on age (Wexler, 1978; Woulfe et al., 2018) and sex (Wexler and Greenberg, 1979) of the animals. Mortality in old males was substantially higher than in young males (Wexler, 1978), while pathophysiology of cardiac damage also differed significantly (Wexler, 1978). In a study comparing adult male and female rats, female rats showed better survival and lower cardiac damage than males (Wexler and Greenberg, 1979). Moreover, gonadectomized adult male and female rats showed better survival and superior repair of their damaged hearts compared to their intact sex-matched controls (Wexler and Greenberg, 1979), supporting an important role of sex hormones. Although authors measured several circulating substances, including adrenocortical hormones, they did not investigate changes in the brain nor behavioral consequences. Behavioral studies, to our knowledge, were only performed in (according to their body weights < 300 g) young male rats (Tkachenko et al., 2018; Hu et al., 2020; Ravindran et al., 2020). Therefore, in the present study, effects of isoproterenol on the heart, neuroinflammation, neuronal function and aspects of behavior were compared in old male and female rats. We hypothesized that the inflammatory response due to high dose of isoproterenol would be reflected in the brain as neuroinflammation, resulting in altered neuronal function with behavioral consequences. Since sex dimorphism is reported for the response on isoproterenol, we anticipate different cardiac and brain responses in old male versus female rats.

## Materials and methods

### Subjects

Twenty-one male and twenty-two female Wistar rats of 24 months age were recruited from our own breeding colony (Semmelweis University, Budapest Hungary). Rats were group housed in a conventional animal facility (22 ± 2°C and humidity of 50 ± 10%). Light was provided from 6 a.m. to 6 p.m. CEST. Standard rodent chow (LT/R, Innovo Ltd., Gödöllõ, Hungary) and tap water were provided *ad libitum*. In case of male rats, 3 rats had to be housed individually due to aggressive behavior. Cleaning of the cages took place 2–3 times per week and was done by an animal caretaker. Bodyweight was measured twice a week. Experimental protocols were approved by the local animal committee of the University of Physical Education, Budapest, Hungary.

### Experimental groups and protocol

**FIGURE 1:**
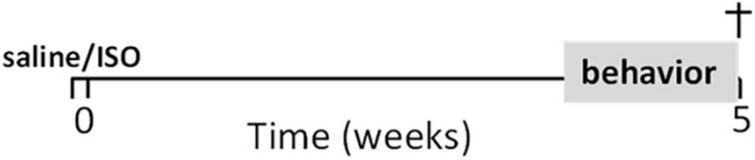
Experimental protocol. ISO, isoproterenol.

Male (*n* = 21) and female rats (*n* = 22) were randomly divided into two groups regarding type of intervention. ISO groups (11 males and 12 females) received 70 mg/kg isoproterenol dissolved in 1 ml/kg saline, control groups (10 males and 10 females) were given 1 ml/kg saline (Toth et al., 2021). Although usually a higher dosage of ISO is used, 85–100 mg/kg, the 70 mg/kg dose was chosen to balance reliable studying effects of ISO and bias because of too high mortality (Toth et al., 2021). Substances were administered on two consecutive days with 24 h in between via subcutaneous injection (Ravindran et al., 2020). This resulted in 21 (10 saline + 11 ISO) females and 22 (10 saline + 22 ISO) male rats. The experimental protocol is summarized in Figure 1. Male and female 24 months old rats were injected with isoproterenol (ISO) or saline, twice with 24 h in between. Four weeks later, behavioral testing was performed, and rats were subsequently sacrificed to collect heart and brain tissue.

### Behavior

Different aspects of behavior were assessed. Exploratory behavior and anxiety were tested in the open field. Effects on cognition were obtained from short-term memory in the novel object recognition test and the novel location recognition test. All tests were recorded with a digital video camera on memory card. Tests started 4 weeks after ISO/saline intervention.

#### Open field exploration test

For the open field test (OF), rats were placed in a round shaped arena (diameter of 80 cm), that was divided into an inner circle (diameter of 32 cm; center area), and an outer wall area. Rats were given 5 min to freely explore it. After removal of the animals, the arena was cleaned with 70% ethanol. Video recordings were analyzed with Eline software (University of Groningen, Netherlands). The time spent in the center and outer wall areas and number of entering of these areas were measured. More time spent in the wall area and less visits to the center area were regarded a measure for anxiety-related behavior, while total number of movements were seen as exploratory behavior.

#### Novel object- and novel location recognition tests

Effects on cognitive performance were tested using the short-term memory test of object and location. The two memory tests were combined in one protocol (Toth et al., 2021) and took place in a black box of 45*55*50 cm. The animal was placed in the box and stayed there for the whole test procedure. Two sets of objects with similar size, weight and shape were used. Animals were unable to replace and/or move the objects. The two sets were randomly rotated through the procedures. Rats were allowed 3 min to explore the box. Then, two identical objects were placed far apart into the arena. After again 3 min the objects were removed, cleaned and after 1 min, one familiar and one novel object were placed back at the same positions as previously; the novel object recognition test (NOR). Again, after 3 min objects were removed, cleaned, and after 1 min the objects were reintroduced with one object placed at a new location on the opposite site of the box; the novel location recognition test (NLR). The test was ended 3 min after exploration of the last setting. Arena and objects were cleaned with 70% ethanol after each animal. Video recordings were analyzed with Eline software (University of Groningen, Netherlands). Time spent exploring the objects was measured. Preference for the novel object or familiar object on a novel place was calculated as time spent on the novel or relocated objected divided by time spent exploring both objects. Data of rats that showed no interest in the objects were omitted from further analyses of these tests.

### Tissue collection and processing

At the end of the experiment rats were terminally anaesthetized with 6% sodium pentobarbital solution injected intraperitoneally (2 ml/kg) and perfused with heparinized (1 ml/l) 0.9% saline via the heart, in order to remove blood. Heart and brain tissues were dissected, and fixated in 4% buffered formaldehyde freshly depolymerized from paraformaldehyde. After 4 days, tissue was washed in 0.01 M phosphate buffered saline (PBS), dehydrated using a 30% sucrose solution, and subsequently quickly frozen in liquid nitrogen and stored at –80°C. From the brain, coronal sections (25 μm) were cut and stored as free-floating sections in 0.01 M PBS containing 0.1% sodium azide at 4°C, till processing for immunohistochemical staining to visualize microglia or brain derived neurotrophic factor expression. Transvers sections (25 μm) of heart tissue at mid-ventricular level were paced on glass immediately after cutting, and processed for histochemical staining to measure collagen levels.

### (Immuno)histochemistry

#### Microglia

To visualize microglia, immunohistochemical staining of ionized calcium binding adaptor molecule 1 (IBA-1) was performed, as described in detail previously (Hovens et al., 2014a). Briefly, sections were incubated with 1:2,500 rabbit-anti IBA-1 (Wako, Neuss, Germany), followed by incubation with 1:500 goat-anti rabbit secondary antibody (Jackson, Wet Grove, United States). After incubation with avidin-biotin peroxidase complex (Vectastain ABC kit, Vector, Burlingame, United States), labeling was visualized by diaminobenzidine (DAB). Sections were mounted onto glass slides. Photographs were taken from the prefrontal cortex-PrL (PFC) and the dorsal hippocampus (DH; CA1, CA3, Dentate Gyrus and Hilus areas) at 200 times magnification. Microglia morphology was analyzed by image analyses (Image Pro plus, United States), according to our previous publication (Hovens et al., 2014a), regarding coverage, density, cell size, cell body area and processes area. Microglia activity was calculated as cell body area/total cell size. Values for the overall dorsal hippocampus were obtained from the average of the values of the different hippocampal areas.

#### Brain-derived neurotrophic factor

Brain Derived Neurotrophic Factor (BDNF) antibody was used to obtain BDNF expression in the PFC and the different areas of the DH; CA1, CA3, Dentate Gyrus and Hilus. Sections were incubated with this first antibody with 1:1,000 dilution (Alomone Labs, Israel), followed by the incubation with 1:500 goat-anti rabbit secondary antibody (Jackson, Wet Grove, United States). Similar to IBA-1, sections were incubated with avidin-biotin peroxidase complex and were visualized by DAB. BDNF expression was measured as corrected optical density (Image-J) compared to an underlying reference area was used as final outcome measure (Hovens et al., 2014b).

#### Cardiac collagen

Heart sections were stained with Sirius red (Sigma-Aldrich) and fast green as counterstaining (Hovens et al., 2016). Percentage collagen was used to measure cardiac damage. Color pictures were taken (20×) to visualized the whole left ventricle. Image analysis (Image Pro plus, United States) was used to measure the collagen positive (red) area and was expressed as percentage of total left ventricular tissue area.

### Data analysis

Data are presented as mean ± standard error of the mean (S.E.M), unless indicated otherwise. Results outside twice the standard deviation of its group were considered outliers and were excluded before analyses (maximally 2 per experimental group). Results were compared using two-way analysis of variance (ANOVA) with least square difference (LSD) *post-hoc* test, with male/female and saline/ISO as factors. Association between selected parameters were analyzed with Pearson linear correlation. For the NOR/NLR tests, outcomes were also tested against change level (= 50%), using a single sample *t*-test. *P*-values of < 0.05 were considered statistically significant and presented as * or ^#^. Potentially relevant tendencies (*p* < 0.1) were mentioned as well.

## Results

### General

For this experiment 21 male and 22 female Wistar rats were used. From the 11 injected male rats, 4 died within the first 24 h following the first injection and 2 additional premature death occurred later. From the 12 injected female rats, 2 died after the first injection, which this was similar to the spontaneous death in both control saline-treated groups (2 out of 10 rats each).

Male rats weighed significantly more than female rats (*p* < 0.001), irrespective of treatment. Whereas relative heart weight appeared slightly higher in females than in males (*p* = 0.060), relative brain weight was significantly higher in females (Table 1). No significant effects of ISO, nor interaction between sex and treatment were observed.

**TABLE 1:** Bodyweight at sacrifice and relative organ weight in the experimental groups.

	Male saline (*n* = 8)	Male ISO (*n* = 5)	Female saline (*n* = 6)	Female ISO (*n* = 10)
Body weight (g)	438 ± 33	416 ± 37	289 ± 12*	276 ± 14*
Heart weight (%body weight)	0.33 ± 0.03	0.35 ± 0.04	0.38 ± 0.01	0.40 ± 0.02
Brain weight (%body weight)	0.50 ± 0.04	0.52 ± 0.05	0.69 ± 0.02*	0.63 ± 0.07

ISO = isoproterenol. *Significant difference between corresponding female and male groups (p < 0.05). Bold values refer to statistically significant effects.

### Cardiac collagen

**FIGURE 2:**
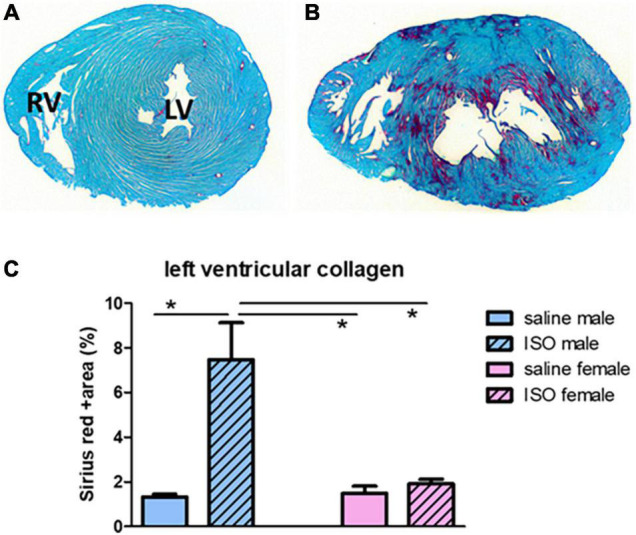
Illustration of transversal sections of the heart with Sirius red staining of cardiac collagen. Upper part shows the result of collagen staining (red) in a saline treated male rat **(A)** and an ISO treated male rat **(B)**. LV, left ventricle; RV, right ventricle. Lower graph **(C)** actual measurements of collagen percentage in the different experimental groups. ISO, isoproterenol. *Significant difference between indicated groups (*p* < 0.05).

Percentage of collagen in the left ventricle at midventricular levels was obtained to measure cardiac damage. Figure 2 illustrates collagen deposition (red) in saline and ISO treated male rats. According to the two-way ANOVA statistics, a significant effect of male/female, saline/ISO, as well as an interaction effect could be observed. However, in more detail (*post hoc* analyses), saline treated males did not differ from saline treated females, and ISO only increased collagen in male rats.

### Neuroinflammation

**FIGURE 3:**
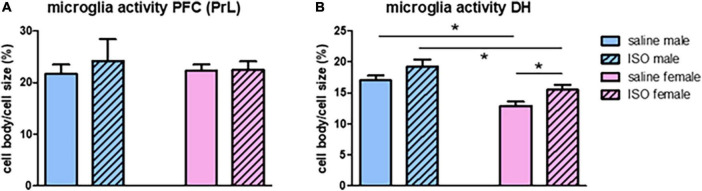
Microglia activity in the prefrontal cortex (PFC; **A**) and the dorsal hippocampus (DH; **B**) in the different experimental groups (*n* = 8,5,7,10 in saline male, ISO male, saline female and ISO female rats, respectively). ISO, isoproterenol. *Significant difference between indicated groups.

Neuroinflammation was measured as microglia activity, obtained from morphological changes. In the prefrontal cortex, no sex-related differences, nor effects of ISO were observed in microglia activity (Figure 3A). In contrast, in the hippocampus, female rats had significantly lower microglia activity than males; significant sex-effect. Also, a significant effect of ISO was seen, which was most pronounced in female rats (Figure 3B).

**FIGURE 4:**
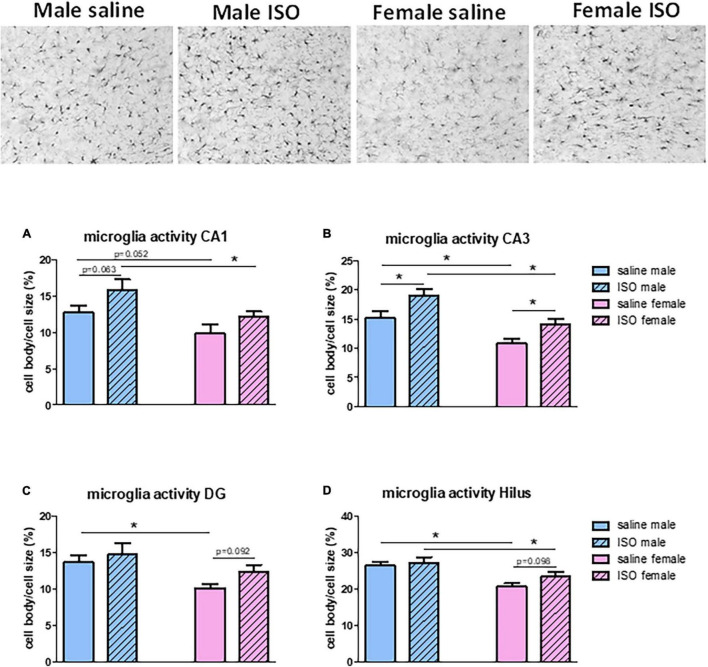
Top: typical pictures of microglia in the hippocampal CA3 area of rats from the different experimental groups (200×). **(A–D)** Measurements of microglia activity in the different areas of the hippocampus, CA1, CA3, dentate gyrus (DG) and Hilus, in the different experimental groups (*n* = 8,5,7,10 in saline male, ISO male, saline female and ISO female rats, respectively). ISO, isoproterenol. *Significant difference between indicated groups. Relevant tendencies with *p* < 0.1 are presented as well.

Results in the hippocampus have been studied in more detail regarding the different hippocampal areas. Effects of overall hippocampal microglia activity (Figure 3B); significant sex differences and significant effects of ISO, were best reflected in the CA3 area (Figure 4 top pictures and panel Figure 4B), whereas in the other areas (Figures 4A,C,D), sex differences (significant) were more prominent than potential effects of ISO (non-significant) (Figure 4), with no interaction between these factors.

The underlying morphologic parameters of the microglia in these areas are presented in Table 2. In most areas, significant differences were observed between the sexes and/or ISO or saline treatment (two-way ANOVA), but no interactions between these factors were found. In all, but the Hilus, ISO increased the microglia cell body size in male as well as in female rats. Although in these areas processes size was consistently lower after ISO, it did not reach statistical significance. In the CA3, females showed lower density and larger microglia compared to male rats; the latter attributable to increased processes.

**TABLE 2:** Morphological parameters of microglia in the different hippocampal areas, CA1, CA3, dentate gyrus (DG) and Hilus, in the different experimental groups.

Experimental group/Brain area	Male saline (*n* = 8)	Male ISO (*n* = 5)	Female saline (*n* = 7)	Female ISO (*n* = 10)
**CA1**				
Density (#/area)	46.9 ± 2.7	49.5 ± 2.5	40.2 ± 3.2	42.8 ± 2.0
Coverage (%)	12.1 ± 0.4	11.9 ± 0.9	13.5 ± 0.8	12.5 ± 0.5
Cell size (pixel)	2,896 ± 422	2,543 ± 284	3,702 ± 424	3,096 ± 222
Cell body size	317 ± 7	363 ± 6*	318 ± 13	343 ± 6*
Processes size	2,580 ± 425	2,180 ± 286	3,384 ± 422	2,753 ± 220
**CA3**				
Density (#/area)	59.4 ± 3.9	62.6 ± 1.8	**50.0 ± 3.2^#^**	**52.2 ± 2.4^#^**
Coverage (%)	11.9 ± 4.1	11.7 ± 6.9	13.1 ± 7.0	12.5 ± 5.4
Cell size (pixel)	2,139 ± 208	1,896 ± 77	**2,900 ± 140^#^**	**2,512 ± 197^#^**
Cell body size	299 ± 6	349 ± 5*	294 ± 12	326 ± 4*^#^
Processes size	1,840 ± 210	1,547 ± 79	**2,606 ± 144^#^**	**2,186 ± 198^#^**
**DG**				
Density (#/area)	55.7 ± 3.1	54.3 ± 1.8	48.8 ± 1.7	51.5 ± 2.4
Coverage (%)	14.4 ± 1.2	13.5 ± 0.8	15.1 ± 0.6	14.1 ± 0.4
Cell size (pixel)	2,964 ± 632	2,583 ± 243	3,284 ± 202	2,894 ± 216
Cell body size	312 ± 6	346 ± 8*	300 ± 9	329 ± 4*
Processes size	2,651 ± 633	2,238 ± 244	2,985 ± 201	2,565 ± 217
**Hilus**				
Density (#/area)	101.5 ± 5.8	97.7 ± 5.7	86.5 ± 4.5	93.3 ± 3.9
Coverage (%)	12.8 ± 0.5	12.8 ± 0.9	13.4 ± 0.6	13.5 ± 0.5
Cell size (pixel)	1,294 ± 36	1,319 ± 38	**1,586 ± 56^#^**	1,487 ± 91
Cell body size	333 ± 6	353 ± 17	316 ± 11	331 ± 4
Processes size	961 ± 38	966 ± 40	**1,269 ± 54^#^**	1,156 ± 90

ISO, isoproterenol. *Significant effect of ISO compared to sex-matched saline (p < 0.05). ^#^Significant difference between sexes with the same treatment (ISO/saline) (p < 0.05). Bold values refer to statistically significant effects.

### Neuronal function

**FIGURE 5:**
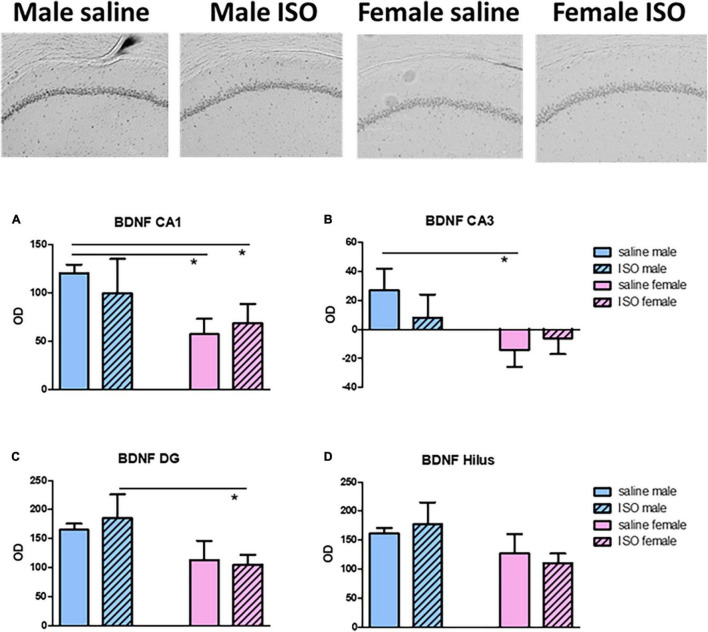
Top: typical pictures of the Brain-derived neurotrophic factor (BDNF) staining in the CA1 area of the different experimental groups. **(A–D)** BDNF expression, expressed as relative optical density (OD), in the different areas of the hippocampus, CA1, CA3, dentate gyrus (DG) and Hilus, in the different experimental groups (*n* = 8,5,6,10 in saline male, ISO male, saline female and ISO female rats, respectively). ISO, isoproterenol. *Significant difference between indicated groups.

Brain-derived neurotrophic factor (BDNF) expression was used as an indicator for neuronal function in the prefrontal cortex and hippocampus. Whereas in the prefrontal cortex, a tendency for declined expression in ISO treated rats was observed (*p* = 0.068), in the dorsal hippocampus, females showed a significantly lower BDNF expression than male rats. When looked into more detail to the different hippocampal areas (Figure 5), the above tendency was substantiated in all but the Hilus area.

### Behavior

**FIGURE 6:**
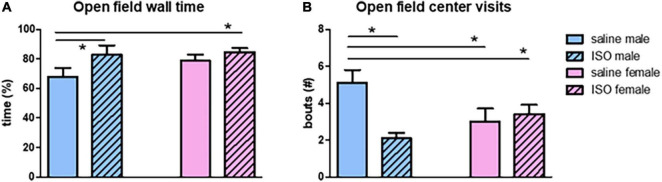
Behavior in the open field test in the different experimental groups (*n* = 8,7,7,10 in saline male, ISO male, saline female and ISO female rats, respectively). ISO, isoproterenol. *Significant difference between indicated groups (*p* < 0.05). **(A)** Time spent at the wall; **(B)** number of visits to the center.

Effects on behavior were studied using exploration in the OF test and short-term memory in the NOR and NLR tests. In the OF, time spent at the wall showed significant sex differences; male rats spent more time in this area (Figure 6A). Similarly, male rats paid significantly less visits to the center area (Figure 6B). No significant effects of ISO were observed. However, a significant interaction between the two factors, sex and treatment, for both parameters indicated that male rats became more anxious after ISO, whereas females did not.

For the NOR/NLR tests, data of several rats had to be excluded as these rats did not show interest in the objects, resulting in reduced numbers of rats per group with high variation (Table 3). Consequently, results from these short-term memory tests did not reveal any statistical effects of sex and/or ISO, nor interaction (Table 3). In fact, only female rats with ISO treatment performed significantly about chance level in the NOR test (indicated by #).

**TABLE 3:** Outcome of short-term memory tests in the different experimental groups.

	Male saline	Male ISO	Female saline	Female ISO
Novel object preference (%)	62 ± 7 (*n* = 8)	78 ± 18 (*n* = 4)	63 ± 13 (*n* = 7)	63 ± 3 (*n* = 6)#
Novel location preference (%)	51 ± 10 (*n* = 6)	72 ± 10 (*n* = 5)	67 ± 11 (*n* = 7)	63 ± 10 (*n* = 9)

ISO, isoproterenol. ^#^Significantly different from change level (= 50%); p < 0.05.

## Discussion

### General

Acute sympathetic stress in patients can be mimicked by high dose of isoproterenol in rats. Animal studies indicated age- and sex associated peripheral effects. The isoproterenol-associated peripheral inflammatory response could be reflected in the brain as neuroinflammation with consequently behavioral changes. So far behavioral changes are only sparsely investigated, and only in young male rats. Therefore, the aim of the present study was to investigate effects of isoproterenol in old male and female rats, regarding survival, cardiac damage, neuroinflammation, neuronal function and behavior.

Main findings were that aged male rats suffer more from ISO than old female rats. Male rats showed higher mortality, more cardiac damage and more anxiety/depressive-like behavior after ISO. Baseline hippocampal microglia activity and brain function were lower in females than in males. ISO induced significant neuroinflammation, but that appeared most pronounced in female rats. ISO did not distinguish between the sexes regarding neuronal function. In conclusion, indeed sex dimorphic effects of ISO were observed, with old male rats being more susceptible than old females. However, these sex dimorphic ISO effects seemed not reflected in differences in neuroinflammation or brain function.

### Effects of isoproterenol in aged rats

In 24 months old male rats, but not in female rats, ISO induced cardiac fibrosis, as seen by the increased collagen percentage 5 weeks later. The mechanism underlying the ISO-induced cardiac damage is widely investigated (reviewed by Nichtova et al., 2012). Briefly, ISO increased heart rate and contractility, causing an imbalance between oxygen demand and consumption, thereby irreversibly damaging cardiomyocytes. This process evokes wound healing and scar formation, with increased levels of cytokines (Alemasi et al., 2019), ultimately resulting in cardiac hypertrophy and heart failure. In the present study, only old rats have been included, hampering direct comparison with younger ones to establish an age effect. For the latter we have to rely on comparing present results to those obtained in our studies in adult 12 months old male (Toth et al., 2021) and female rats (under review for publication), showing that ISO caused significant cardiac damage in both male and female rats. Although in the 12 months old male rats, microglia morphology suggested ISO-induced hippocampal neuroinflammation, BDNF expression was not affected, and OF behavior even suggested lower anxious/depressive-like behavior, suggesting interaction between the time courses of age-related and ISO-related processes. The increased cytokine levels may be reflected in the brain as neuroinflammation. Several studies support the release of proinflammatory cytokines shortly after ISO treatment (Alemasi et al., 2019; Li et al., 2021; Al-Botaty et al., 2022). Moreover, the cytokines that appeared increased in plasma after ISO, TNFα, IL6, and IL1β (Li et al., 2021), are also the ones shared between depression and heart failure (Pasic et al., 2003). Specifically, TNFα may play a role in leakage of the blood brain barrier (Liu et al., 2013; Al-Botaty et al., 2022). Although this short-term inflammatory response may subsequently subside, its reflection in the microglia in the brain may be prolonged as neuroinflammation. Indeed, in the present study, ISO induced hippocampal neuroinflammation 5 weeks later. A direct effect of beta-adrenergic stimulation on microglia priming has been demonstrated (Johnson et al., 2013), but effects that persists for weeks after acute peripheral ISO administration has not been reported before. No significant effects of ISO were demonstrated on BDNF expression, indicating no effect on neuronal function. In the behavioral tests, specifically male ISO-treated rats may become more anxious. Similarly, depressive-like behavior was reported 7 weeks after ISO (Hu et al., 2020). Moreover, comparable to our NOR and NLR test outcomes, no effects on working memory 8 days after ISO were observed, but long-term learning and memory seemed impaired after ISO (Ravindran et al., 2020). However, effects on cognitive performance in the present study may remain inconclusive, because of low number of successful tests and high variability. In general, in the present study, similar effects of ISO were observed as seen in the literature in young male rats. However, substantial sex-related differences could be distinguished.

### Sex dimorphism in isoproterenol response

ISO induced substantial mortality in male rats, whereas similar doses in female rats did not exceed mortality in saline treated rats. This is in general agreement with literature (Wexler, 1978). Five weeks after ISO, cardiac collagen was still increased in male-, but not in female rats. Similarly, chronic ISO stimulation causing fibrosis in male but not female rat hearts was reported, which seemed attributable to different responses of cardiac fibroblasts (Peter et al., 2021). Male cardiac fibroblasts were more activated than female cardiac fibroblasts, consistent with elevated fibrosis in male rat hearts, and may be attributed to higher β-adrenoceptor expression and phosphokinase-A activation in male fibroblasts (Peter et al., 2021). The observation that gonadectomized male and female rats showed better survival and superior repair of their damaged hearts compared to their intact sex-matched controls (Wexler and Greenberg, 1979), supported an important role of sex hormones.

Consistent with these findings, male rats, but not female rats, showed more anxious behavior. Although this anxious/depressive-like behavior has been reported before in literature (Hu et al., 2020), no sex dimorphism was investigated here. Moreover, all behavioral studies on the effects after ISO-induced cardiac damage were only performed in young male rats (Tkachenko et al., 2018; Hu et al., 2020; Ravindran et al., 2020). However, our previous study in rats with myocardial infarction induced by coronary artery ligation also showed behavioral changes in male, but not in female rats (Gouweleeuw et al., 2016).

We hypothesized that the ISO-induced peripheral inflammatory response would be reflected in the brain as neuroinflammation, potentially leading to declined neuronal functioning and consequently behavior changes (Quan, 2014). From above observations, male rats would be anticipated to display more neuroinflammation and further declined BDNF expression than female rats. However, BDNF expression was lower in females than in males, and not significantly affected by ISO. Indeed, male rats showed higher control microglia activity in the hippocampus than female rats, but the responses to ISO were comparable in both sexes. Moreover, these responses were best reflected in the CA3 area of the hippocampus, an area involved in pattern separation (Stevenson et al., 2020), rather than in the CA1 area involved in learning and memory (Stevenson et al., 2020). This would be consistent with the lack of differences in the short-term memory (NOR and NLR) tests. Therefore, the effects of ISO that were predominantly seen in male rats, could not be directly attributed to differences in neuroinflammation or neuronal function in the investigated brain areas. Other mechanisms may be involved, including glucose- and lipid metabolism (Wexler and Greenberg, 1979). Nevertheless, the older studies of Wexler and coworkers (Wexler, 1975; Wexler and Greenberg, 1979) clearly indicated sex differences in the response of peripheral parameters that could be linked to behavioral changes, such as depression and cognition (Keller et al., 2017). For instance, the corticosterone response during the first week after ISO appeared much higher in female rats than in male rats (Wexler and Greenberg, 1979), suggesting different hypothalamic–pituitary–adrenal (*HPA*) *axis* activity. Compared to males, female rats show a more robust HPA axis response, as a result of circulating estradiol levels which elevate stress hormone levels during non-threatening situations as well as after stressors (Oyola and Handa, 2017), with distinct behavioral consequences (reviewed by Packard et al., 2016).

### Limitations

In the present study, only old rats have been included, hampering direct comparison with younger ones to establish an age effect. Comparison with results from our studies in 12 months old rats (Toth et al., 2021) may indicate interaction between ISO-induced effects and age-related processes. Although selected carefully, effects were studied at single time points, limiting the overview of time courses of these ongoing processes.

In accordance with the lack of ISO-induced cardiac fibrosis in old female rats, the increased ISO-induced mortality in male rats was absent in female rats. For the male rats, but not for the females, this could have led to selection of the “better” old rats for analyses. Analyses of survivors may have led to underestimation of effects, hence biasing proper comparison between the sexes.

On the other hand, since ISO was dosed based on body weight, female rats with significantly lower body weights and higher relative heart (ns) and brain weights, could have received too less ISO to cause an effect. However, in our previous studies in 12 months old male (Toth et al., 2021) and female rats (Toth et al., 2022), using the same dose per body weight as used in the present study, led to a significant and similar magnitude of cardiac damage and reduced cardiac function in both sexes.

Finally, in hindsight, collecting blood samples to measure circulating (inflammatory) factors could have further elucidated on the mechanism of the inflammation/neuroinflammation process.

## Conclusion

Our data indicated that old male rats appeared more susceptible to ISO than old female rats, by displaying higher mortality, more cardiac damage and more anxious/depressive-like behavior, indeed supporting sex dimorphism in ISO responsiveness. Since ISO did not distinguish between the sexes regarding microglia activation or BDNF expression, mechanisms other than neuroinflammation and/or altered neuronal function seemed to underly this sex dimorphism.

## Data availability statement

The original contributions presented in this study are included in the article/supplementary material, further inquiries can be directed to the corresponding author.

## Ethics statement

The animal study was reviewed and approved by Animal Ethical Committee of University of Physical Education, Budapest, Hungary.

## Author contributions

KT, RS, EZ, and CN contributed to the conception and design of the study. KT and TO performed the animal experiment. KT and RS analyzed and organized the data. RS did the statistical analysis and wrote the first draft of the manuscript. All authors contributed to the final version of manuscript and approved the final version.

## Conflict of interest

The authors declare that the research was conducted in the absence of any commercial or financial relationships that could be construed as a potential conflict of interest.

## Publisher’s note

All claims expressed in this article are solely those of the authors and do not necessarily represent those of their affiliated organizations, or those of the publisher, the editors and the reviewers. Any product that may be evaluated in this article, or claim that may be made by its manufacturer, is not guaranteed or endorsed by the publisher.
